# Phylogenetic analysis and temporal diversification of mosquitoes (Diptera: Culicidae) based on nuclear genes and morphology

**DOI:** 10.1186/1471-2148-9-298

**Published:** 2009-12-22

**Authors:** Kyanne R Reidenbach, Shelley Cook, Matthew A Bertone, Ralph E Harbach, Brian M Wiegmann, Nora J Besansky

**Affiliations:** 1 Eck Institute for Global Health, Department of Biological Sciences, University of Notre Dame, Notre Dame, IN 46556, USA; 2 Department of Entomology, The Natural History Museum, Cromwell Road, London, SW7 5BD, UK; 3 Department of Entomology, Gardner Hall, North Carolina State University, Raleigh, NC 27695, USA

## Abstract

**Background:**

Phylogenetic analyses provide a framework for examining the evolution of morphological and molecular diversity, interpreting patterns in biogeography, and achieving a stable classification. The generic and suprageneric relationships within mosquitoes (Diptera: Culicidae) are poorly resolved, making these subjects difficult to address.

**Results:**

We carried out maximum parsimony and maximum likelihood, including Bayesian, analyses on a data set consisting of six nuclear genes and 80 morphological characters to assess their ability to resolve relationships among 25 genera. We also estimated divergence times based on sequence data and fossil calibration points, using Bayesian relaxed clock methods. Strong support was recovered for the basal position and monophyly of the subfamily Anophelinae and the tribes Aedini and Sabethini of subfamily Culicinae. Divergence times for major culicid lineages date to the early Cretaceous.

**Conclusions:**

Deeper relationships within the family remain poorly resolved, suggesting the need for additional taxonomic sampling. Our results support the notion of rapid radiations early in the diversification of mosquitoes.

## Background

Mosquitoes (Diptera: Culicidae) are a monophyletic group of true flies [1-4], recognizable by their elongate adult mouthparts through which the females of most species feed on vertebrate blood. Mosquitoes occur throughout temperate and tropical regions, and well beyond the Arctic Circle, but are most diverse in tropical forest environments [5]. A bewildering amount of morphological diversity parallels their spectacular radiation into virtually every conceivable collection of water, ranging from a few droplets trapped by plant parts to large bodies of fresh and brackish surface water, making mosquitoes "as ubiquitous as water" [6]. The relationship between human health and those species that are medically important (<200 of 3,524 currently recognized; http://mosquito-taxonomic-inventory.info/) has driven most mosquito research. Within this small subset of disease vector species, morphological similarities between close relatives (*e.g*., cryptic or sibling species complexes) continue to pose practical and academic challenges to disease control, conventional taxonomy, and phylogenetic inference. Ironically, it is not the morphological similarity but rather the morphological diversity of mosquitoes that has confounded efforts to delimit many supraspecific groups and reconstruct their evolutionary history.

The Culicidae had an ancient origin, probably in the Jurassic [7,8], consistent with the fossil record of their sister group Chaoboridae [9]. Unfortunately, the sparse mosquito fossil record sheds no light on evolutionary relationships in the family. Traditional classification of Culicidae based on the phenetic framework of Edwards [7] resulted in arbitrary groupings reflecting intuitive interpretation of morphological similarities. More modern classifications have incorporated important revisions of select genera and tribes based on explicit methodology, but in the absence of a comprehensive application of quantitative methods across the family, the result still does not entirely reflect evolutionary history [reviewed in [2]]. Current classification divides the family into two subfamilies (Anophelinae and Culicinae), 11 tribes, and a minimum of 44 genera [2,10-13] (Table 1).

**Table 1:** Species of Culicidae included in the phylogenetic analysis, with reference to their classification and distribution [after 2].

Subfamily Tribe	Genus	**No. Subg**.	No. spp.^1^	Distribution	Species studied
*Anophelinae*	*Anopheles*	7	455	Cosmopolitan	*Anopheles gambiae *Giles
					*Anopheles atroparvus *van Thiel
	*Bironella*	3	8	Australasian	*Bironella gracilis *Theobald
	*Chagasia*	-	4	Neotropical	
Culicinae					
Aedeomyiini	*Aedeomyia*	2	6	Afrotropical, Australasian, Oriental, Neotropical	*Aedeomyia squamipennis *Lynch Arribálzaga
Aedini^2^	*Aedes*	23	363	Old World, Nearctic	*Aedes aegypti *Linnaeus
	*Armigeres*	2	58	Australasian, Oriental	*Armigeres subalbatus *(Coquillett)
	*Ayurakitia*	-	2	Oriental	
	*Borichinda*	-	1	Oriental	
	*Eretmapodites*	-	48	Afrotropical	*Eretmapodites quinquevittatus *Theobald
	*Haemagogus*	2	28	Principally Neotropical	*Haemagogus equinus *Theobald
	*Heizmannia*	2	39	Oriental	
	*Ochlerotatus*	22	550	Cosmopolitan	*Ochlerotatus triseriatus *(Say)
	*Opifex*	-	1	New Zealand	*Opifex fuscus *Hutton
	*Psorophora*	3	48	New World	*Psorophora ferox *(von Humboldt)
	*Udaya*	-	3	Oriental	
	*Verrallina*	3	95	Principally Australasian, Oriental	
	*Zeugnomyia*	-	4	Oriental	
Culicini	*Culex*	23	763	Cosmopolitan	*Culex quinquefasciatus *Say
	*Deinocerites*	-	18	Principally Neotropical	
	*Galindomyia*	-	1	Neotropical	
	*Lutzia*	3	7	Afrotropical, Australasian, Oriental, Neotropical, eastern Palaearctic	
Culisetini	*Culiseta*	7	37	Old World, Nearctic	*Culiseta inornata *(Williston)
Ficalbiini	*Ficalbia*	-	8	Afrotropical, Oriental	
	*Mimomyia*	3	44	Afrotropical, Australasian, Oriental	*Mimomyia luzonensis *(Ludlow)
Hodgesiini	*Hodgesia*	-	11	Afrotropical, Australasian, Oriental	
Mansoniini	*Coquillettidia*	3	57	Old World, Neotropical	*Coquillettidia perturbans *(Walker)
	*Mansonia*	2	23	Old World, Neotropical	
Orthopodomyiini	*Orthopodomyia*	-	38	Afrotropical, Nearctic, Neotropical, Oriental, Palaearctic	*Orthopodomyia alba Baker*
Sabethini	*Isostomyia*	-	4	Neotropical	
	*Johnbelkinia*	-	3	Neotropical	
	*Kimia*	-	5	Oriental	
	*Limatus*	-	8	Neotropical	*Limatus durhami *Theobald
	*Malaya*	-	12	Afrotropical, Australasian, Oriental	*Malaya genurostris *Leicester
	*Maorigoeldia*	-	1	New Zealand	*Maorigoeldia argyropus *(Walker)
	*Onirion*	-	7	Neotropical	
	*Runchomyia*	2	7	Neotropical	
	*Sabethes*	5	38	Neotropical	*Sabethes cyaneus *(Fabricius)
	*Shannoniana*	-	3	Neotropical	*Shannoniana fluviatilis *Lane and Cerquiera
	*Topomyia*	2	54	Principally Oriental	
	*Trichoprosopon*	-	13	Neotropical	*Trichoprosopon digitatum *(Rondani)
	*Tripteroides*	5	122	Principally Australasian, Oriental	*Tripteroides bambusa *(Yamada)
	*Wyeomyia*	15	140	Principally Neotropical	*Wyeomyia smithii *(Coquillett)
Toxorhynchitini	*Toxorhynchites*	4	88	Afrotropical, Australasian, Neotropical, eastern Palaearctic, Oriental	*Toxorhynchites amboinensis *(Doleschall)
Uranotaeniini	*Uranotaenia*	2	265	Afrotropical, Australasian, Oriental, Neotropical	*Uranotaenia sapphirinia *(Osten Sacken)

^1^For the current number of valid species recognized in generic level taxa of the family, consult http://mosquito-taxonomic-inventory.info/

^2^Generic-level classification of Aedini predates Reinert et al. [10], except for *Borichinda*.

Generic-level relationships across all Culicidae have rarely been studied. The first attempt [14] was based on comparative bionomics and morphology using intuitive methods typical of that time. Surprisingly, further attempts using modern cladistic methods were not made for nearly 50 years. The most comprehensive of these phylogenetic re-analyses employed 73 morphological characters to examine the relationships of the 38 genera then recognized [1]. In general, almost none of the hypotheses raised by the 1951 phylogeny were supported in the 1998 reconstruction, with few exceptions including the monophyletic and basal position of the subfamily Anophelinae, and the monophyly of the tribes Sabethini and Culicini. However, most characters were homoplastic - some extensively - and many relationships were inadequately resolved. Although the Harbach and Kitching [1] study challenged traditional generic groupings and reinforced the need for reappraisal, it also suggested that robust recovery of generic-level relationships of Culicidae may be difficult with morphological characters alone.

Only four higher-level phylogenies of Culicidae based on gene sequences have been published, each of which were very taxon-limited in scope. All were able to show that, in agreement with the morphological phylogenies, *Anopheles *was sister to other sampled genera [4,15-17]. The study by Miller et al. [4], based on four mosquito species, was inconclusive and the more comprehensive study of Shepard et al. [15], based on 18S rDNA sequences of 39 species representing nine genera, was unable to resolve deeper relationships. Of the four studies, only Besansky and Fahey [16] employed a single-copy nuclear protein coding gene to assess relationships. Their study, based on the *white *gene, included 13 species representing nine genera. When third codon positions were excluded, Anophelinae was recovered as a basal lineage and Sabethini, Culicini and Aedini were recovered as monophyletic, suggesting the potential of protein-coding sequences for reconstructing generic-level relationships within Culicidae.

The importance of sampling multiple genes when attempting to reconstruct species phylogenies is well recognized [18]. Mitochondrial DNA and nuclear ribosomal DNA are convenient targets, due to conserved primer binding sequences and ease of amplification based on their typically high copy number. However, both can be problematic for resolving deep phylogenetic relationships. Mitochondrial DNA may exhibit a high mutation rate, skewed base composition, and even symbiont-induced biases [19,20]. Beyond base compositional bias, ribosomal DNA also can be exceedingly difficult to align [21]. Given the increasing availability of completely sequenced mosquito genomes, protein-coding nuclear genes represent a viable alternative as well as a rich and largely untapped resource.

In the study reported here, we explored the phylogenetic utility of six nuclear protein-coding genes: *arginine kinase*, CAD, *catalase*, *enolase*, *hunchback*, and *white*. As noted above, only *white *was used previously in mosquitoes [16]. All except *catalase *have been used in other insect groups: CAD in bees, empidoid flies, and lacewings, among others [22-24]; *arginine kinase *in hymenopterans [25,26]; *enolase *in beetles [27]; and *hunchback *in Hawaiian drosophilids [28]. We sequenced these six genes from 26 mosquito species representing 25 genera, and two chaoborid outgroup species. In addition, 80 morphological characters were scored from these mosquito and outgroup species. Our goals were twofold: (1) to estimate a generic-level phylogeny of Culicidae based on molecular and morphological evidence, and (2) to use fossils and sequence data to infer divergence times for major culicid lineages.

## Methods

### Taxon sampling

Twenty-six species of mosquitoes representing 25 genera were used as ingroup taxa (Table 1). Two chaoborid midges, *Eucorethra underwoodi *and *Chaoborus astictopus*, were used as outgroup taxa based on the sister-group relationship between Chaoboridae and Culicidae [3,29,30]. Specimens were preserved in 70-100% ethanol at -20°C. Sampling of additional ingroup genera for this study was precluded by unavailability of specimens adequately preserved for molecular analysis; DNA from pinned museum specimens or specimens stored at room temperature for prolonged periods was found to be excessively degraded.

### DNA extraction, PCR, and sequencing

**Figure 1:**
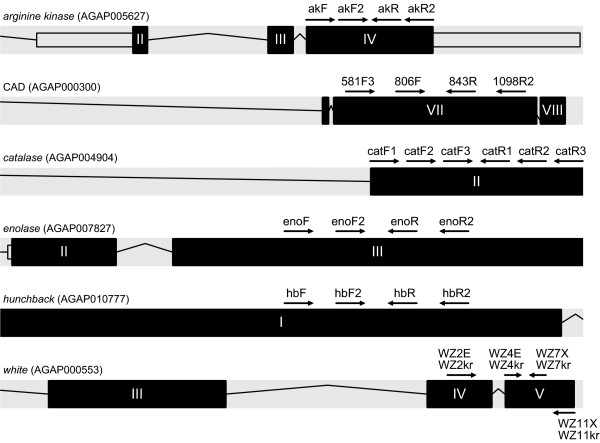
**Approximate location and orientation of primers (black arrows) with respect to their target exons, in the context of gene models (or portions thereof) based on the *An. gambiae *genome**. Models were exported from the Transcript View available in VectorBase http://www.vectorbase.org; protein-coding exons are indicated by black rectangles with Roman numerals; introns by black lines. Gene names and corresponding *An. gambiae *gene ID (AGAP number) are provided at top left. Not to-scale.

Sequences were obtained from six nuclear protein-coding genes (*arginine kinase*, CAD, *catalase, enolase, hunchback*, and *white*) from VectorBase http://www.vectorbase.org for the three mosquito species with completely sequenced genomes (*Anopheles gambiae*, *Culex quinquefasciatus*, and *Aedes aegypti*), from GenBank for available *white *sequences (GenBank accession numbers U73829, AF318199, AF318200, U73834, U73827, AF318206, U73835, U73837, AY055811, AF318193, AF318209), or by PCR amplification and direct sequencing. Genes were amplified and sequenced using degenerate primers previously reported in the literature, or designed based on amino acid alignments of respective genes from the three sequenced mosquito species. Primer sequences and their sources are provided in Table 2. Primers were located in regions that would allow amplification within a single large exon, or across exons separated by small introns, to facilitate amplification from genomic DNA templates. Primers were hemi-nested, whereby the first round of amplification based on the outermost pair was followed by alternative second round PCR from which two internal, overlapping fragments were amplified (Figure 1).

**Table 2:** Primers used in this study.

Gene	Primer	Sequence (5'-3')^1^	Source, if not this study
*arginine kinase*	akF	GCTTCAAGAAGACCGACAAGCAC	
	akF2	AAGACCTTCCTGGTCTGGTGC	
	akR	ACCCWKCTGCATSGAGATGATG	
	akR2	GCCATCGTACATCTCCTTGACG	
CAD	581F3	AAYCCIAAYATYGCIACIGTICARAC	
	806F	GTNGTNAARATGCCNMGNTGGGA	[23]
	843R	GCYTTYTGRAANGCYTCYTCRAA	[23]
	1098R2	CAICCIACIGCRCACCARTCRAAYTC	
*Catalase*	catF1	ACTTYGACCGKGAGCGIATTCC	
	catF2	GGTTTCGCYSTSAARTTCTACAC	
	catF3	GAYGGYTWYCGITTCATGAACG	
	catR1	GCCYTGRTYIGWYTTGAAGTGGAAC	
	catR2	GAASGARTTSGGRWAGTAGTTSG	
	catR3	GRCGKCCRAARTCRGCATCAAC	
*Enolase*	enoF	ATGCAGGAGTTCATGATCCTG	
	enoF2	GTACGATCTGGACTTCAAGAAC	
	enoR	TCCTGGTCRAAGGGATCCTC	
	enoR2	AGRATYTGGTTGTACTTGGC	
*hunchback*	hbF	ACICCICCIATGGAYGTIACICCICC	
	hbF2	TGYCCIAARTGYCCITTYGTIACIG	
	hbR	TGRCARTAYTTIGTIGCRTARTTRC	
	hbR2	GCYTGYTGRTCIGCRAACATYTGRA	
*white*	WZ2E	(E)AAYTAYAAYCCIGCIGAYTTYTA	[65]
	WZ2kr	AYTAYAAYCCIGCIGAYTTYTAYG	
	WZ4E	(E)GGIGTIATGAAYATHAAYGG	[65]
	WZ4kr	GAYGGIGTIATGAAYATHAAYGG	
	WZ7X	(X)TCRAAIACRTTYTCRAAIGTCATR	[53]
	WZ7kr	GCRAAIACRTTYTGRAAIGTC	
	WZ11X	(X)TTIARRAARAAICCICCRAA	[65]
	WZ13kr	GCYTCRTTIGCRTAICKRAACC	

^1^Degeneracy indicated by the IUB code; I is inosine; linkers (in parentheses) are X, an *XbaI *linker: CGTCTAGA, and E, an *EcoRI *linker: GGAATTC.

Genomic DNA was extracted from whole mosquitoes using the DNeasy kit (Qiagen, Valencia, CA). PCR reactions (50 μl) contained 20 mM Tris-HCl (pH 8.4), 50 mM KCl, 1.5 mM MgCl_2_, 200 μM each dNTP, 2.5 U Taq polymerase, 2.4% DMSO, 0.5% BSA, 25 pmol each primer, and 1 μl template DNA (~1/50^th^-1/500^th ^of the amount extracted from a single mosquito). Amplifications consisted of 1 cycle at 93°C for 1 min; 35 cycles of 94°C for 20 sec, 45°C for 20 sec, and 72°C for 2 min; and a final extension cycle of 72°C for 5 min. After inspection of an aliquot by electrophoresis through a 1.5% agarose gel, products of the expected size were excised from the gel and purified using GeneClean Kit (MP Biomedicals, Irvine, CA), or sequenced directly if only one band was observed. Excess primers and dNTPs were removed by adding 2 U of exonuclease 1, 1 U of shrimp alkaline phosphatase, and 1.8 μl of H_2_O to 8 μl of PCR product, incubating at 37°C for 15 min, and inactivating at 80°C for 15 min before sequencing. Sequencing was carried out using an Applied Biosystems 3730 × l DNA Analyzer and Big Dye Terminator v3.1 chemistry. Electropherograms were inspected and trimmed using SeqMan II (DNASTAR, Madison, WI). Sequences were deposited in GenBank under accession numbers: *arginine kinase*, GQ906806-GQ906829; CAD, GQ906830-GQ906854; *catalase*, GQ906855-GQ906874; *enolase*, GQ906879-GQ906901; *hunchback *GQ906902-GQ906926; *white*, GQ906927-GQ906937.

### Morphological characters

Morphological structures were examined in the adult, pupal and larval (fourth-instar) stages. Heads were removed from adult mosquitoes for comparative studies of structures not readily visible in intact specimens. Heads were cleared in 5% sodium hydroxide solution, stained in acid fuchsin and mounted frontodorsal side uppermost in Euparal on microscope slides. Pinned adults were examined under simulated natural light. Dissected genitalia, larvae, and larval and pupal exuviae were studied with differential interference contrast optics. The morphological terminology follows Harbach and Knight [31,32] and Harbach and Kitching [1].

The 28 species were coded for 80 characters (see additional file 1: FileS1) derived from fourth-instar larvae (24), pupae (12), adults (36), female genitalia (1), and male genitalia (7). The data matrix is shown in Additional file 2: TableS2. Characters were coded from direct observations except in a few cases where structures were missing from available specimens. Some missing data for the immature stages of *Chaoborus astictopus *were coded from literature sources. Characters that could not be scored due to missing data or absence of homologous structures (e.g., character 20 in three anophelines and two chaoborid species that lack a siphon) were denoted by a "?". All multistate characters were treated as unordered. Most of the structures and states of characters are illustrated in Harbach & Knight [31] and Harbach & Kitching [1].

### Phylogenetic analysis

Nucleotide sequence alignments were guided by the corresponding amino acid alignments, using utilities within the program suite EMBOSS [33]; http://embossgui.sourceforge.net/demo/. Inferred amino acid sequences were aligned using the program "emma," which provides an interface to ClustalW [34]. The Gonnet matrix [35] was chosen and the resulting alignment was followed by limited manual adjustment. "Tranalign" was then used to align the nucleotide sequence based on the previously aligned amino acid sequence. Introns were removed from aligned gene regions before analysis.

Basic sequence information (pairwise sequence divergence, base composition, statistical tests of homogeneity of base composition, number of variable and parsimony informative characters) was obtained using PAUP* v4.0b10 [36]. In addition, plots of transitions and transversions versus divergence at each codon position were based on observed (uncorrected) p-distances from PAUP*.

Maximum parsimony (MP) analyses were implemented in PAUP* on a phylogenetic data set that included concatenated genes with/without morphological characters. Third codon positions for each gene were removed and gaps were treated as missing data. Heuristic searches consisted of 1000 random sequence additions with tree bisection-reconnection (TBR) branch swapping. Bootstrap support values were based on 500 replicates, each with 10 random additions and TBR branch swapping.

Maximum likelihood (ML) analyses were performed on molecular data sets only, which included both individual/concatenated genes, with/without third codon positions. The ML heuristic searches were performed in PAUP*, using the model of nucleotide substitution and parameter values selected via Modeltest [37]. Values for the substitution matrix, base composition, gamma distribution of among-site rate variation (G) and the proportion of invariant sites (I) are available from the authors on request. Bootstrap resampling was conducted using 1000 replicate neighbour-joining (NJ) trees based on the ML substitution matrix. The Shimodaira-Hasegawa test [[38]; data not shown] was used to test for incongruence between phylogenies suggested by individual genes, or successive combinations of congruent genes.

Bayesian (BI) phylogenetic tree searches were performed in MrBayes 3.1.2 [39] on concatenated gene and gene + morphology data sets using aligned nucleotides, both including and excluding third codon positions, and using concatenated aligned amino acids. For concatenated genes (nucleotides) and genes (nucleotides) + morphology, a mixed model approach was used with model parameters specified per gene partition according to Modeltest and a Markov K + G model for morphology, with branch lengths unlinked and estimated for each partition [40,41]. Bayesian tree searches using aligned amino acids were carried out in MrBayes 3.1.2 using the WAG model of amino acid evolution (WAG+I+G) [42]. Each Bayesian search was carried out for 10,000,000 generations (sampling every 1000) using four chains (default heating parameters) and a 30% burn-in value. The included Bayesian sets of trees were sampled after likelihood scores reached convergence and the mean split difference values were below 0.02.

### Divergence time estimation

Estimates of divergence times for mosquito lineages were calculated using the parametric Bayesian-relaxed clock approach implemented in the programs ESTBRANCHES and MULTIDIVTIME [43] and using the combined gene data set (nucleotides) including third codon positions. Branch lengths and evolutionary rate priors were estimated from the data using the BASEML program in the PAML software package [44] and ESTBRANCHES. Tree topology, minimum and maximum root node age, and fossil-based minimum age constraints are set as user-defined analysis priors. For the tree topology we used the tree recovered from the BI search of combined amino acids (see *Phylogenetic Analyses*, below). The root max-min age prior between Culicidae and outgroups was set as 230-187 Ma corresponding to the hypothesized age of the Diptera [6,8] and a fossil assignable to the Chaoboridae [187 Ma; ref [9]], and three lineages were constrained according to fossil-based minimum ages (*Toxorhynchites mexicanus*, 16 Ma; *Culex winchesteri*, 34 Ma; *Anopheles dominicanus*, 34 Ma; http://mosquito-taxonomic-inventory.info/category/fossil-culicidae/fossil-culicidae)[45]. We followed the analytical procedure described in Rutschmann et al. [46] and in the MULTIDIVTIME readme files, and ran the Markov chain for 1.1 × 10^6 ^cycles with samples collected every 100 cycles and discarded the first 100,000 cycles as burn-in. We performed the MULTIDIVTIME analysis multiple times from different initial conditions to confirm convergence of the Markov chain on highly similar resulting time estimates and posterior intervals.

## Results and Discussion

### Sequence variation

**Figure 2:**
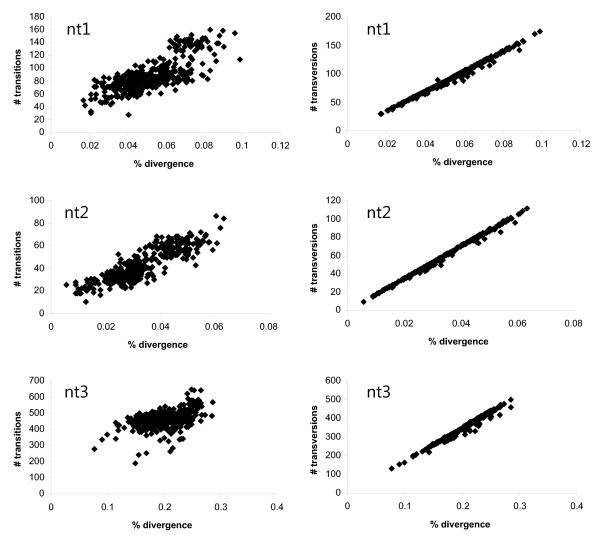
**Numbers of transitions or transversions at each codon position (nt1, nt2 and nt3) plotted against uncorrected nucleotide divergence for pairwise species comparisons across the combined six-gene data set**.

Across six genes, the molecular data matrix consisted of 5352 aligned characters, of which 2839 were variable and 2259 were parsimony informative (Table 3). Not surprisingly, most of the variation was found in the third codon (nt3) position. Analysis of base composition of the combined genes for each major taxonomic grouping revealed significant departures from homogeneity at the nt3 position, owing to three groups: Anophelinae, Culicini (*Cx. quinquefasciatus*) and Ficalbiini (*Mimomyia luzonensis*) (Table 4). Moreover, plots of transitions and transversions against uncorrected pairwise nucleotide divergences at each codon position suggested saturation of transitions at the nt3 position (Figure 2). These results prompted us to perform ML and BI phylogenetic analyses both with and without the nt3 partition, and to exclude this partition in MP analyses (see Phylogenetic analyses).

**Table 3:** Character information for genes used in this study.

			Parsimony informative			
			
	Aligned	Variable	All	nt1	nt2	nt3
*arginine kinase*	717	270	203	32	14	157
CAD	1467	767	667	131	68	468
*catalase*	753	398	350	72	43	235
*enolase*	699	287	232	33	15	184
*hunchback*	951	682	444	101	82	261
*white*	765	435	363	84	47	232
All genes	5352	2839	2259	453	269	1537

**Table 4:** GC content and compositional heterogeneity of major taxonomic groupings based on the species analyzed.

	Total	nt1	nt2	nt3
Anophelinae	62.1*	57.9	40.8	87.5*
Aedeomyiini	54.2	57.5	39.1	68.1
Aedini	51.6	53.7	38.2	63.2
Culicini	57.5*	54.5	38.7	79.3*
Culisetini	51.5	53.7	38.4	62.9
Ficalbiini	55.1*	52.9	39.0	73.9*
Mansoniini	50.4	52.8	38.8	59.8
Orthopodomyiini	52.6	53.5	38.9	65.6
Sabethini	50.6	52.9	38.7	60.5
Toxorhynchitini	50.8	53.7	38.2	60.7
Uranotaeniini	52.1	53.1	38.6	64.8
Chaoboridae	47.4	50.5	38.4	53.3

*χ*^2^	991.37	96.56	31.66	2271.27
*P*-value	0.00	0.11	1.00	0.00

Uncorrected pairwise sequence divergence within the mosquitoes sampled ranged widely, from 10-27% (summarized by major taxonomic groupings in Table 5). However, the average distance between tribes and subfamilies was at the upper end of the range (20% and 23%, respectively), approaching that between mosquitoes and their sister group, the chaoborid midges (26%).

**Table 5:** Mean pairwise uncorrected *p*-distances (%) across all six genes.

Taxonomic grouping	% (min-max)
Between Families	
Culicidae-Chaoboridae	26.4 (23.9-30.2)
Between subfamilies	
Anophelinae-Culicinae	22.5 (17.8-27.4)
Within Anophelinae	13.1 (12.2-14.2)
Within Culicinae	19.8 (10.0-27.4)
Between Tribes	19.7 (11.9-25.5)
Within Tribes	
Aedini	16.6 (10.0-21.1)
Sabethini	16.6 (13.6-18.8)

### Phylogenetic analyses

Evidence was found for incongruence among some genes or gene combinations via the Shimodaira-Hasegawa test [38]; data not shown. However, examination of phylogenies resulting from individual genes revealed that topological incongruence was generally limited to certain poorly supported nodes. It has been suggested that different data sets may have a common phylogenetic signal recoverable only upon combined analysis [47,48], and, under the hypothesis that combining data from multiple genes may potentially overcome misleading signal in individual genes [49], we conducted further analyses to compare results from a concatenated data set with results obtained from ML and BI.

**Figure 3:**
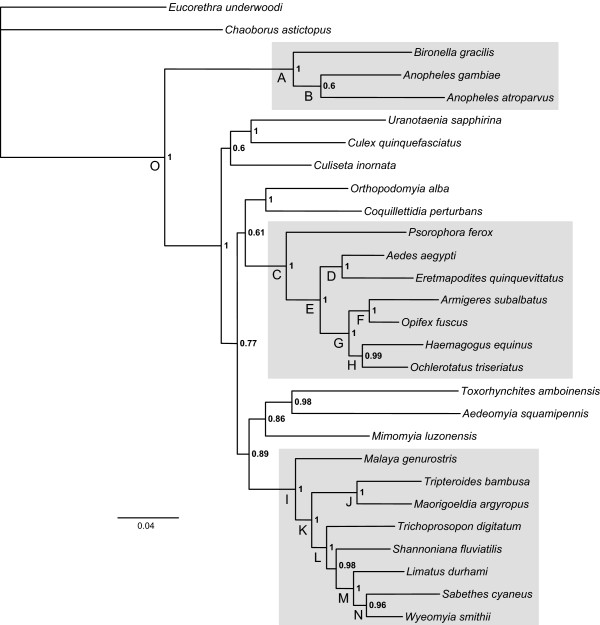
**Phylogram of relationships among mosquito species, inferred by Bayesian likelihood analysis of combined amino acids**. Amount of inferred character change is indicated by the scale bar below. Numbers associated with nodes are Bayesian posterior probabilities above 0.5. Letters associated with nodes refer to bootstrap support values and posterior probabilities estimated from alternative analyses, provided in Table 6. Gray-shaded boxes enclose (from top to bottom) Anophelinae, Aedini and Sabethini.

Relationships inferred by MP and ML, including BI, are summarized in Figure 3 and Table 6. All three algorithms, as applied to various data partitions (± nt3; ± morphological characters; nucleotides or amino acids), gave overwhelming support for the monophyly of Culicidae (node O), the monophyly and basal position of the subfamily Anophelinae (node A; gray box), and the monophyly of the tribe Sabethini (node I; gray box). Less conclusive support by ML, but reasonable support by MP and BI, was observed for the monophyly of the tribe Aedini (node C). These results confirm the conclusions of Harbach [2] regarding what was already known about the phylogeny of mosquitoes.

**Table 6:** Bootstrap support or posterior probabilities for relationships inferred within Culicidae based on combined gene sequences, with or without morphological characters. Nodes refer to Figure 3.

	Maximum parsimony		Maximum likelihood		Bayesian				
			
Node	Mol (-nt3)	Mor+Mol (-nt3)	Mol (+nt3)	Mol (-nt3)	Mol (+nt3)	Mor+Mol (+nt3)	Mol (-nt3)	Mor+Mol (-nt3)	AA
**A**	100	100	100	100	1.0	1.0	1.0	1.0	1.0
**B**	71	64	100	83	1.0	1.0	---	---	0.6
**C**	58	73	65	51	1.0	1.0	1.0	1.0	1.0
**D**	62	59	93	51	1.0	1.0	0.74	0.8	1.0
**E**	100	100	100	100	1.0	1.0	1.0	1.0	1.0
**F**	54	51	98	64	---	---	0.51	---	1.0
**G**	55	51	92	58	---	---	0.51	---	1.0
**H**	87	89	100	74	1.0	1.0	1.0	1.0	0.99
**I**	99	97	100	100	1.0	1.0	1.0	1.0	1.0
**J**	100	100	100	100	1.0	1.0	1.0	1.0	1.0
**K**	63	60	69	62	---	---	0.98	0.6	1.0
**L**	92	90	100	98	1.0	1.0	1.0	1.0	1.0
**M**	68	81	100	98	1.0	1.0	1.0	1.0	1.0
**N**	---	---	100	96	1.0	1.0	---	---	0.96
**O**	100	100	100	100	1.0	1.0	1.0	1.0	1.0

Mol, molecular characters; Mor, morphological characters; ± nt3, third nucleotide position included or excluded; AA, amino acid sequences.

Support varied for relationships within these well-supported clades. The subfamily Anophelinae was represented in this study by three species from two genera: *Bironella *(*Bironella gracilis*) and *Anopheles *(*Anopheles atroparvus*, subgenus *Anopheles*; *An. gambiae, subgenus Cellia*). Not all analyses supported the monophyly of the genus *Anopheles*; an alternative relationship of *Bi. gracilis + An. atroparvus *was also recovered. There is precedence for the paraphyly of *Anopheles *relative to *Bironella *in previous morphological [50,51] and molecular [52] studies. Reliable inference of relationships between these groups may be problematic due to conflicting signals or contemporaneous radiations, but the suggestion of Sallum et al. [50] to redefine *Bironella *as an informal group within *Anopheles *seems premature [2,53].

Within tribe Sabethini, *Malaya *occupied the most basal position among the taxa sampled, although this placement was not recovered in a subset of BI analyses. The genus *Maorigoeldia*, containing only a single species exclusive to New Zealand, was sister to *Tripteroides *(Oriental, Australasian and Palaearctic species), in all cases with 100% bootstrap support or posterior probability of 1.0. These relationships were not recovered in cladistic analyses of morphological data that included representatives of a larger number of genera. Belkin [54] regarded *Maorigoeldia *to be sister to all other sabethine species. This was supported in the studies of Harbach and Kitching [1], Harbach and Peyton [55], and Harbach et al. [56], but not in the study of Judd [57], which placed *Maorigoeldia *as the sister group to the New World genera of Sabethini. Whereas *Malaya *was recovered as the sister of genus *Topomyia *in the first two of these four studies, it was paired with *Limatus *in the most derived clade of Sabethini when Harbach et al.[56] included the new genus *Kimia *in the data set of Harbach and Peyton [55]. Also, *Tripteroides *(Old World) was recovered as sister to *Trichoprosopon *(New World), which is supported by shared morphological characters that are unique to these two genera.

Decisive support for monophyly of the New World genera (*Limatus*, *Sabethes*, *Shannoniana*, *Trichoprosopon*, and *Wyeomyia*) was found, in agreement with previous studies [2,57]. Among these genera, a close relationship between *Limatus, Sabethes *and *Wyeomyia *was strongly supported by all analyses, but other nodes were unstable. Although not apparent in Figure 3, *Shannoniana *and *Trichoprosopon *showed a sister relationship in all but the BI amino acid analysis. However, as indicated in the previous paragraph, the results of cladistic analyses based on morphological data and a larger sample of sabethine taxa casts doubt on these relationships.

The remarkably large tribe Aedini (1255 species, http://mosquito-taxonomic-inventory.info/taxonomy/term/6065) has been the subject of recent efforts to infer higher-level relationships based on morphological characters of all life stages [10-13]. Although this has resulted in major changes to classification, phylogenetic resolution has been limited. In the present study, as in the cladistic analyses of extensive morphological data by Reinert et al. [11-13], *Psorophora *was recovered as sister to all other Aedini. Sister-group relationships strongly supported in most cases were *Aedes *(*Stegomyia*) + *Eretmapodites *and *Haemagogus *+ *Ochlerotatus*. Other relationships within Aedini were less clear. Moreover, no consensus could be reached regarding affinities of any other genera within Culicidae as a whole, outside of Aedini and Sabethini.

### Divergence time estimates

**Figure 4:**
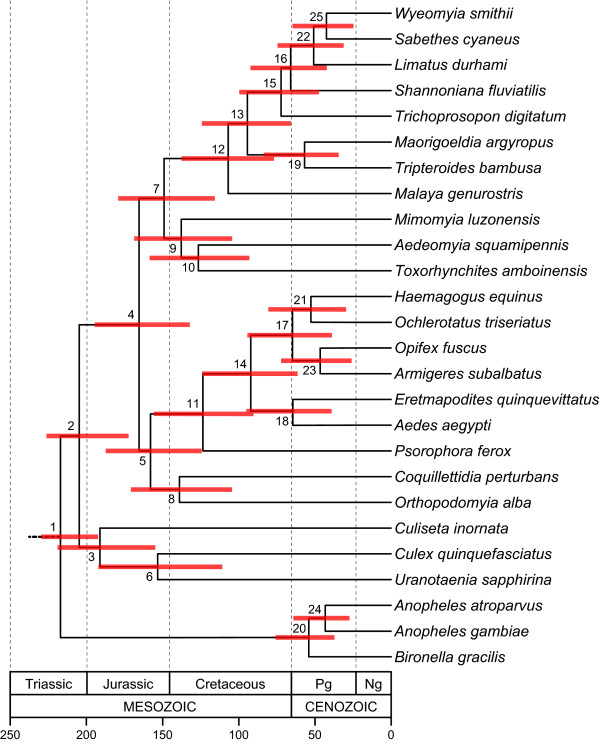
**Chronogram of mosquito age divergences with 95% confidence intervals (red bars) **Numerical node ages and their 95% confidence intervals are presented in Table 7. Calibration points: Chaoboridae+Culicidae, 230-187 Ma [8,9]; *Toxorhynchites mexicanus *fossil, ≥ 16 Ma [66]; *Culex winchesteri *fossil, ≥ 34 Ma [67]; *Anopheles dominicanus *fossil, ≥ 34 Ma [45].

A chronogram for Culicidae is given in Figure 4, and corresponding divergence time estimates are provided in Table 7. Based on the taxa sampled and three fossil constraints, earliest divergence within mosquitoes - between the lineages leading to Culicinae and Anophelinae - dates to ~226 Ma. This estimate is in reasonable agreement with Krzywinski et al. [58], who determined the split between *Anopheles *and *Aedes *(*Stegomyia*) to have occurred ~145-200 Ma based on mitochondrial DNA sequences. Although 226 Ma is substantially older than the 118 Ma divergence between Chaoboridae and Culicidae estimated by Bertone et al. [8], the 95% credibility ranges overlap between studies. Because the latter study was aimed at deeper divergences within lower Diptera, Bertone et al. [8] only included two mosquitoes and one chaoborid, possibly accounting for the discrepancy. Moreover, Bertone et al. [8] estimated divergence times from a single gene (28S rDNA) and used only a few fossil calibration points (none close to Culicidae), which may also have contributed to differences in age estimates. We favor the older divergence estimates, as they are consistent with other evidence suggesting that mosquitoes likely originated in the Jurassic [7,59]. As early as 1923, Edwards [60] surmized that the "origin and phylogenetic history of the Culicidae must go back to well into the Mesozoic Era."

**Table 7:** Divergence time estimates and credibility intervals (Ma) for nodes in Figure 4.

Node	Time (Ma)	CI (Ma)	Node	Time (Ma)	CI (Ma)
1	216.86	229.50 - 192.19	14	92.05	123.87 - 61.07
2	204.53	226.22 - 172.28	15	72.31	99.71 - 47.39
3	191.04	218.81 - 154.68	16	66.00	92.37 - 42.28
4	165.31	194.41 - 132.01	17	64.60	94.29 - 38.72
5	157.79	187.17 - 124.13	18	64.44	94.85 - 38.93
6	153.07	191.95 - 110.61	19	56.69	83.49 - 34.25
7	148.94	179.01 - 115.70	20	53.94	75.76 - 37.14
8	138.75	170.62 - 104.19	21	52.59	80.56 - 29.45
9	137.56	168.53 - 104.14	22	50.89	74.61 - 31.19
10	126.38	158.42 - 92.81	23	46.36	72.08 - 25.80
11	123.39	155.71 - 90.18	24	43.11	63.95 - 27.20
12	106.86	137.35 - 76.70	25	42.51	64.85 - 24.73
13	94.20	124.15 - 65.57			

Unexpectedly, the split between *Anopheles *and *Bironella *was a remarkably shallow 51 Ma. This estimate contradicts Krzywinski et al. [58], who estimated a substantially greater (90-106 Ma) divergence between the *Anopheles *subgenera, *Anopheles *and *Cellia*, represented in this study by *An. atroparvus *and *An. gambiae*, respectively. To what extent this incongruence can be explained by limited taxon sampling or biases in the molecules used to infer divergence dates (*e.g*., strong base composition bias in Anophelinae; Table 4), is presently unknown. However, given the universal agreement that *Anopheles *occupies an early-branching position among the Culicidae, it seems likely that our crown-group estimates do not accurately reflect the age of this group.

The only well-supported clades of the subfamily Culicinae in the phylogenetic analyses are the tribes Aedini and Sabethini, which apparently arose at similar times (roughly 112 and 115 Ma, respectively) and diversified more recently. Eight genera of the subfamily Culicinae (*Aedeomyia*, *Coquillettidia*, *Culiseta*, *Culex*, *Mimomyia*, *Orthopodomyia*, *Toxorhynchites*, *Uranotaenia*), whose relationships were not strongly or consistently recovered, represent the deeper internal branches of the tree. The nodes connecting these branches are not only ancient (exceeding 127 Ma), but also relatively close together in time, occurring within a ~30 million year interval between 127-158 Ma. If these estimates are corroborated in the future by denser taxon sampling and more fossil-based age constraints, they will support the notion of rapid radiations early in the diversification of mosquitoes, potentially explaining the difficulty in attaining a stable phylogeny for these lineages. An early Cretaceous timing for these rapid radiations is consistent with the appearance of angiosperms, a group of plants whose nectar is exploited as an energy source by mosquitoes [61], and whose water-filled parts are the sole habitats occupied by the immature stages of many groups of mosquitoes, notably members of the tribe Sabethini [54].

## Conclusion

This study represents one of the few attempts to reconstruct generic-level relationships within Culicidae as a whole, and the only attempt to combine morphological data and molecular characters from multiple genes. Among molecular phylogenetic studies of the family, it more than doubled the number of taxa sampled to date. Yet results were mixed. The ability to recover previously known clades (Anophelinae, Sabethini, and Aedini) was encouraging. However, the deeper relationships among genera could not be resolved unambiguously, potentially due to ancient and rapid radiation, as hypothesized for other insect groups [6]. There has been much debate regarding whether better resolution and support of relationships is achieved through broader taxonomic sampling [62,63] or sequencing of more loci [64]. The current explosion of sequencing whole genomes from organisms, including mosquitoes, promises many potentially informative genes beyond those included here. On the other hand, the recent study by Wiegmann et al. [49] successfully resolved even deeper divergences and a longstanding controversy in the phylogeny of holometabolous insects, using only six single-copy nuclear genes comprising a similar number of base pairs to that compiled for the present study. Although more molecular, as well as morphological, characters may well prove useful, there is little doubt that broader taxonomic sampling is now the key roadblock. Considering that the mosquito diversity housed in museums is almost invariably preserved in a fashion that has impeded conventional molecular data collection, this roadblock may be substantial. There is an urgent need for fresh museum collections, particularly from under-sampled yet high-biodiversity regions worldwide, and their cryo- or ethanol preservation with vouchers. The present study was limited by what was available in existing collections. Broader taxon sampling is crucial not merely because it may help break up long branches. To the extent that the current generic system of classification includes paraphyletic and polyphyletic groups containing numerous species, it is clear that inclusion of only one or few generic exemplars can be misleading, and that more representative sampling is needed.

## Abbreviations

BI: Bayesian; Ma: million years ago; ML: maximum likelihood; MP: maximum parsimony; nt: nucleotide; PCR: polymerase chain reaction.

## Authors' contributions

NJB and REH conceived and designed the experiments. KRR designed PCR primers and collected molecular data. REH collected morphological data. KRR, SC, MAB and BMW performed experiments and analyzed data. REH contributed specimens. KRR and NJB wrote the paper with contributions by all authors.

## Supplementary Material

Additional File 1 **File S1**. Morphological characters used in the analysis Click here for file

Additional File 2 **Table S2 **Morphological data matrix for 28 taxa and 80 species of Culicidae Click here for file
