# Supplementary material for: Phylogenetic analysis and temporal diversification of mosquitoes (Diptera: Culicidae) based on nuclear genes and morphology
Source: BMC Evol Biol. 2009 Dec 22;9:298. doi: 10.1186/1471-2148-9-298 (PMC2805638; doi:10.1186/1471-2148-9-298)
Supplement: Additional File 1 — File S1. Morphological characters used in the analysis [file 1471-2148-9-298-S1.PDF]

FileS1. Morphological characters used in the analyses.

Larvae (fourth-instars)

1. *Hypostomal suture*: (0) short to absent, not reaching posterior tentorial pit; (1) complete to posterior tentorial pit.

2. *Occipital foramen*: (0) circular to oval; (1) transverse and slit-like.

3. *Maxillary palpus*: (0) appended to maxillary body; (1) fused with maxillary body.

4. *Hypostomal sclerite*: (0) part of lateralia; (1) narrowly attached to lateralia; (2) detached from lateralia (Fig. 2C–G).

5. *Hypostomal sclerite and maxillary palpus*: (0) separate; (1) fused.

6. *Hypostomal sclerite and maxillary body*: (0) separate; (1) fused.

7. *Apical process of maxilla*: (0) absent; (1) present.

8. *Maxillary brush*: (0) absent; (1) composed of independent spicules; (2) represented by a solid claw-like structure without evidence of individual elements.

9. *Seta 2-C*: (0) absent; (1) present.

10. *Seta 3-C*: (0) present, on oral surface of head; (1) present, on adoral surface of head.

11. *Seta 13-P*: (0) absent; (1) present.

12. *Seta 8-M*: (0) absent; (1) present.

13. *Seta 1 on some or all of abdominal segments I-VII*: (0) with flattened leaf-like branches (palmate); (1) with normal stem-like branches.

14. *Setae 6,7-I,II*: (0) single main stem with numerous regularly arranged branches arising on either side (plumose); (1) one or more main stems without plumose branching.

15. *Seta 12-I*: (0) absent; (1) present.

16. *Seta 5-VIII*: (0) removed from seta 4, well below level of dorsal margin of segment X; (1) close to seta 4, usually near or above level of dorsal margin of segment X.

17. *Comb*: (0) absent; (1) present.

18. *Comb plate*: (0) absent or weakly developed; (1) present, well developed.

19. *Siphon* (degree of development): (0) absent; (1) lobe with a narrow posterior band of sclerotized cuticle; (2) short tube with separate anterior and posterior sclerites; (3) elongate fully sclerotized tube.

20. *Seta 1-S*: (0) inserted at base of siphon; (1) inserted beyond base of siphon.

21. *Pecten*: (0) absent; (1) composed of spines; (2) composed of filaments.

22. *Accessory setae of siphon* (*other than 1,2-S*): (0) absent; (1) present.

23. *Saddle*: (0) absent; (1) incomplete; (2) complete, forming a ring around segment X.

24. *Pairs of seta 4-X*: (0) absent; (1) one pair; (2) 4 pairs; (3) usually ≥5 pairs.

Pupae

25. *Dorsal apotome*: (0) evenly sclerotized, appearing as a single sclerite; (1) weakly sclerotized medially, appearing as two sclerites joined by membrane.

26. *Trumpet*: (0) supporting tubercle absent; (1) present.

27. *Tracheoid area of trumpet*: (0) absent; (1) present.

28. *Seta 1-CT* (degree of development): (0) normal, similar in development to setae 2,3-CT; (1) very strongly developed, considerably larger than setae 2,3-CT.

29. *Seta 14-III-VII*: (0) absent; (1) present.

30. *Seta 9-IV-VII*: (0) at or very near caudolateral angle of tergum; (1) removed from caudolateral angle of tergum.

31. *Seta 0-VIII*: (0) inserted on anterior area of tergum; (1) inserted at mid-length or on posterior area of tergum.

32. *Seta 9-VIII*: (0) ventral in insertion; (1) dorsal in insertion; (2) inserted midway between dorsal and ventral surfaces.

33. *Seta 14-VIII*: (0) absent; (1) approximated; (2) widely separated.

34. *Seta 1-IX*: (0) absent; (1) present.

35. *Seta 1-XI*: (0) absent; (1) present.

36. *Paddle seta(e)*: (0) absent; (1) present.

Adults (both sexes except where otherwise indicated)

37. *Erect scales of head*: (0) absent; (1) few and restricted to occiput; (2) numerous and not restricted to occiput.

38. *Interocular space* (principally females): (0) constricted, without scales/setae extending to postfrontal sutures; (1) broader with scales/setae extending to postfrontal sutures.

39. *Interantennal ridge* (females): (0) complete, with dorsal arms reaching ocular sutures and enclosed area without frontal pit; (1) complete, with very short or conjoined dorsal arms reaching postfrontal sutures and engulfing frontal pit (when evident); (2) incomplete in dorsal area of postfrons, with frontal pit reinforced by cuticular ring associated with postfrontal sutures; (3) entirely absent from postfrons, with discrete frontal pit usually removed from postfrontal sutures.

40. *Interantennal ridge* (males): (0) complete in postfrons; (1) incomplete (absent) in postfrons.

41. *Basal microsetae of antennal pedicel*: (0) absent; (1) present.

42. *Apical flagellomeres* (males): (0) one or both (usually) of two apical flagellomeres disproportionately long compared with the other flagellomeres; (1) these flagellomeres not disproportionately long in comparison with the others.

43. *Maxillary palpomeres* (females): (0) five fully developed; (1) four, fifth vestigial or absent; (2) three, fourth vestigial or absent; (3) two, third vestigial if present; (4) one.

44. *Maxillary palpomeres* (males): (0) five; (1) four; (2) three, fourth vestigial or absent; (3) two, third vestigial or absent; (4) one.

45. *Mouthparts*: (0) short, not developed into a proboscis; (1) long, developed into a proboscis.

46. *Labellum*: (0) comprising two separate sclerites; (1) comprising partially (ventrally) fused proximal and distal sclerites; (2) comprising a single sclerite.

47. *Proximal sclerite of labellum*: (0) absent or unrecognizably fused with distal sclerite; (1) short, similar in size to distal sclerite; (2) elongate, distinctly longer than distal sclerite.

48. *Labellar scaling*: (0) absent; (1) present.

49. *Antepronota*: (0) large and approximated; (1) smaller (usually) and more widely separated.

50. *Acrostichal setae*: (0) absent; (1) present.

51. *Dorsocentral setae*: (1) absent; (1) present.

52. *Scutellum*: (0) evenly rounded; (1) trilobed.

53. *Mesopostnotal setae and/or scales*: (0) absent; (1) present.

54. *Paratergite*: (0) bare; (1) with scales or setae.

55. *Postpronotal setae*: (0) absent; (1) present.

56. *Prespiracular setae*: (0) absent; (1) present.

57. *Postspiracular setae*: (0) absent; (1) present.

58. *Prealar setae*: (0) absent; (1) present.

59. *Upper mesokatepisternal setae*: (0) absent; (1) present.

60. *Lower mesepimeral seta(e)*: (0) absent; (1) present.

61. *Metepisternal scales*: (0) absent; (1) present.

62. *Upper calypter*: (0) bare; (1) with one or more setae or hair-like scales.

63. *Vestiture of alula*: (0) absent; (1) present.

64. *Vein R_s with basal spur*: (0) absent; (1) present.

65. *Vein R_2*: (0) shorter than vein R_{2+3}; (1) equal or longer than vein $R_{2+3.}$

66. *Precubital furrow*: (0) absent; (1) present.

67. *Anal vein*: (0) ends before or at junction of mcu and CuA; (1) ends beyond junction of mcu and CuA.

68. *Microtrichia of wing membrane*: (0) minute, inconspicuous at low magnification; (1) distinct, clearly visible at low magnification.

69. *Dorsal tertiary fringe scales/setae* (males): (0) absent; (1) present.

70. *Tarsomere 1 of fore- and midlegs*: (0) shorter than tarsomeres 2-5 combined; (1) longer than tarsomeres 2-5 combined.

71. *Base of hindcoxa*: (0) well below dorsal margin of mesomeron; (1) more or less in line with or slightly above dorsal margin of mesomeron.

72. *Pulvilli*: (0) absent or rudimentary (inconspicuous); (1) distinctly developed (conspicuous).

73. *Spermathecal capsules* (females): (0) one; (1) three.

74. *Genitalia, tergum IX, sclerotization* (males): (0) absent; (1) present.

75. *Genitalia, tergum IX, setae* (males): (0) absent; (1) present.

76. *Genitalia, claspette* (males): (0) absent; (1) lobe at base of gonocoxite; (2) columnar process at base of gonocoxite; (3) basal mesal lobe of gonocoxite; (5) lobe/plaque appressed to mesal surface of gonocoxite; (5) separate basal mesal lobe/plaque; (6) subapical lobe of gonocoxite.

77. *Genitalia, phallosome, aedeagus* (males): (0) two lateral plates; (1) single structure.

78. *Genitalia, phallosome, opisthophallus* (males): (0) absent; (1) present.

79. *Paraprocts* (males): (0) absent or only weakly developed; (1) strongly developed, apex without crown of spicules; (2) strongly developed, apex with crown of spicules.

80. *Genitalia, proctiger, cercal setae* (males): (0) absent; (1) present.
