# Supplementary material for: Phylogenetic analysis and temporal diversification of mosquitoes (Diptera: Culicidae) based on nuclear genes and morphology
Source: BMC Evol Biol. 2009 Dec 22;9:298. doi: 10.1186/1471-2148-9-298 (PMC2805638; doi:10.1186/1471-2148-9-298)
Supplement: Additional File 2 — Table S2 Morphological data matrix for 28 taxa and 80 species of Culicidae [file 1471-2148-9-298-S2.PDF]

TableS2. Morphological data matrix for 28 taxa and 80 species of Culicidae.

	1	1111111112	2222222223	3333333334	4444444445	5555555556	6666666667	7777777778
	1234567890	1234567890	1234567890	1234567890	1234567890	1234567890	1234567890	1234567890
Chaoborus astictopus	1012??0010	0111?0000?	??03011011	1110010110	1000020011	?000100100	0111101110	10111?0000
Eucorethra underwoodi	1010000011	011110002?	0003011011	0111010111	1000000011	1000100010	0110000110	01111?0000
Anopheles gambiae	0002000111	110010001?	1013000010	1211012100	0000100011	1000010110	0101111110	0000011000
Anopheles atroparvus	0002000111	110010001?	1013000010	1211012100	0000110011	1000010110	0101111110	0000011000
Bironella gracilis	0002000111	110010001?	1013000010	1211012100	0100120011	1000000110	0101111110	0000001000
Aedeomyia squamipennis	110???0101	0111101131	0023111010	0021012131	1122101011	1101100111	0110101111	0000030100
Aedes aegypti	1000000101	0111001031	1013101011	0020011111	1020101110	1101101110	0110101100	0011140000
Aedes triseriatus	1001000101	0111101031	1013101011	0020012111	1020101010	1101101110	0110101110	0011120011
Armigeres subalbatus	0002000111	110010001?	1013000011	0020011111	1020101110	0101101111	0110101100	1011141000
Eretmapodites quinquevittatus	1001000101	0111001031	1012100111	0020012111	1020101010	1110101100	0110101100	0011110011
Haemagogus equinus	1001000101	0111101031	1013101111	0020011111	1022101100	0101101100	0110101100	0011121011
Opifex fuscus	1001000101	0111101031	1013100011	0020010110	1122101011	1100101111	0110101110	0011140011
Psorophora ferox	1001000101	0111101131	1023100011	0020012011	1020101011	1100111111	0110101100	0011120011
Culex quinquefasciatus	1001000101	0111101031	1123101011	0021012021	1020101011	1100100111	0110101100	0111161121
Culiseta inornata	1001000101	0111101030	1023100011	0021112011	1010101011	1101110111	0110101100	0011130011
Mimomyia luzonensis	0002000101	0111111031	0022111011	0020012011	1021101111	1100100110	0110001100	0001130011
Coquillettidia perturbans	1001000101	0111111031	0023111011	1120002011	1010101011	1100100111	0110101100	0011130011
Orthopodomyia alba	1002000101	0111101031	0023100011	0020012111	1010101011	1100100110	0110101111	0011130011
Limatus durhami	1102011100	0111001031	0111100101	0020001031	0133102100	0110000100	0011101110	1011151011
Malaya genurostris	1112011100	0111011031	1111100101	0020000131	0133102100	0100110000	1010100110	1011131000
Maorigoeldia argyropus	1002001100	1111011031	1111100111	0020001031	1022101111	1100110110	0111101110	0011131111
Sabethes cyaneus	1112011100	0111011031	2111100101	0020001031	1133102100	0110010000	0011101110	1011151011
Shannoniana fluviatilis	0002001200	0111001031	0111100101	0020001031	1030102110	0110010100	0111101100	1011151011
Trichoprosopon digitatum	1002101100	0011010031	0111100101	0020001031	1030102110	0110110100	0111101100	1011151011
Tripteroides bambusa	1002101100	1111011031	1111100111	0020001031	1033102110	1100110100	0110101100	1011131011
Wyeomyia smithii	1102001100	0111011031	0111100101	0020000031	1133102100	0110010100	0011101110	1011151011
Toxorhynchites amboinensis	1002101100	0011010031	0111100101	0201101021	1010120110	0000010101	0001011100	1011131011
Uranotaenia sapphirina	0002000101	0111101131	1023101011	0221012021	1044101011	1100110111	0010000000	0001030000
